# A testable framework linking diagnostic AI contribution to outcome measurement in clinical decision support

**DOI:** 10.3389/fdgth.2026.1871438

**Published:** 2026-07-07

**Authors:** Jan Kirchhoff, Fabian Berns, Christian Schieder, Johannes Schobel

**Affiliations:** 1 DigiHealth Institute, Neu-Ulm University of Applied Sciences, Neu-Ulm, Germany; 2 medicalvalues GmbH, Karlsruhe, Germany; 3 Weiden Business School, OTH Amberg-Weiden, Weiden, Germany

**Keywords:** AI governance, artificial intelligence, clinical decision support systems, diagnostic AI contribution score, health informatics, large language models, learning health systems, outcome-based metrics

## Abstract

Artificial intelligence-enabled clinical decision support systems (AI-CDSS) are increasingly embedded in diagnostic and therapeutic workflows, yet evaluation, procurement, and governance often remain anchored in model-centric indicators such as discrimination, calibration, sensitivity, and specificity. These indicators are necessary but insufficient for determining whether a deployed AI-CDSS improves patient-relevant outcomes, clinical workflow, resource use, economic performance, clinician and patient experience, learning, and equity. This article proposes a testable conceptual framework linking the Diagnostic AI Contribution Score (DACS) to outcome-domain prioritization and auditable measurement design. The contribution is theory-building and methodological rather than empirically validating: no new patient-level data were generated or analyzed. We conducted a concept-driven structured narrative synthesis across clinical AI evaluation, clinical decision support, health-services research, value-based care, software measurement, AI governance, and large language model (LLM) deployment. We used the synthesis to derive a DACS-informed framework for selecting outcome domains, specifying exposure–action–outcome linkage, and defining minimum telemetry needed for auditability. The revised framework provides four core outputs: (i) a six-domain taxonomy of outcome metrics for AI-CDSS, separating learning/governance from equity; (ii) a comparison with existing AI evaluation and reporting frameworks to clarify the framework’s incremental contribution; (iii) a DACS-to-domain mapping and explicitly illustrative prioritization heuristic, accompanied by a threshold-sensitivity logic; and (iv) a four-step measurement framework with minimum telemetry requirements and clinical implementation guardrails. LLM-specific extensions address provenance and version tracking, human mediation, documentation-quality audits, latency and compute metering, and workflow-mediated effects. We further specify clinical safety considerations including alert fatigue, override patterns by user group, automation bias and diagnostic anchoring, educational effects, de-implementation criteria, fallback workflows, integration with existing quality-management infrastructure, and patient-facing disclosure where appropriate. The proposed framework should be interpreted as a structured, hypothesis-generating model for proportional outcome measurement and governance. Future work should empirically test inter-rater reliability of DACS scoring, validate DACS-to-domain mappings through expert elicitation and prospective deployments, calibrate thresholds across use cases, and evaluate whether standardized telemetry improves attribution, monitoring, and accountability for AI-CDSS.

## 1 Introduction

Across acute care, radiology, pathology, laboratory medicine, chronic disease management, and documentation-intensive clinical processes, artificial intelligence-enabled clinical decision support systems (AI-CDSS) have become part of routine diagnostic and therapeutic workflows [1, 2]. In this article, *AI-CDSS* refers to AI-enabled decision support embedded in clinical workflows, including alerting, triage, diagnostic work-up support, pathway support, documentation support, and patient communication support, irrespective of whether the underlying model is task-specific, predictive, rule-augmented, generative, or foundation-model-based.

Evaluation of AI-CDSS has traditionally emphasized model-centric performance indicators such as area under the receiver operating characteristic curve (AUROC), sensitivity, specificity, calibration, and positive predictive value. These indicators remain necessary for technical and regulatory assessment, but they do not by themselves answer the implementation-relevant question: whether an AI-CDSS changes care delivery, patient outcomes, safety, resource allocation, clinician workload, patient experience, or organizational performance in routine care [3, 4]. This gap is clinically important because the same model can have very different consequences depending on local workflow integration, alert presentation, user expertise, staffing, governance, and data quality [1, 5]. A sepsis model that performs acceptably in retrospective validation may still create excessive alert burden, poor alert-to-action ratios, or unsafe overreliance after deployment [6]. Conversely, a system with modest model-centric gains may generate relevant value if it reduces diagnostic delay, prevents low-yield testing, improves documentation quality, or makes escalation pathways more reliable.

Outcome-based metrics are therefore central to real-world evaluation, post-deployment governance, and adoption decisions. They are also prerequisites for any downstream accountability arrangement, including procurement, coverage, reimbursement, service-level agreements, or outcome-linked contracting [7–9]. However, outcome measurement in AI-CDSS is difficult because outcomes differ in proximity and attributability. Proximal outcomes, such as alert acknowledgment, time to action, documentation time, or test-order changes, are often more directly linkable to AI exposure. Distal outcomes, such as mortality, readmissions, or total episode costs, may be clinically meaningful but require stronger study designs, risk adjustment, and sensitivity analyses before attribution to AI-CDSS can be defended.

Existing reporting and evaluation guidance for clinical AI has substantially improved transparency, trial reporting, early-stage evaluation, and prediction-model appraisal. CONSORT-AI and SPIRIT-AI strengthen reporting of clinical trials involving AI interventions [10, 11]. DECIDE-AI provides guidance for early-stage clinical evaluation of decision-support systems [12]. TRIPOD+AI and PROBAST+AI support reporting and bias assessment for prediction models [13, 14]. Broader translational frameworks such as TEHAI emphasize multi-domain evaluation and implementation context [4]. These frameworks are essential, but they generally do not specify how outcome-domain priorities should vary across concrete AI-CDSS deployment profiles, nor what minimum telemetry is needed to make exposure, action, and downstream outcomes auditable in routine workflows.

This article addresses that gap by proposing a testable conceptual framework that links the Diagnostic AI Contribution Score (DACS) to outcome-domain prioritization and auditable measurement design. DACS was introduced in prior work as a structured characterization of diagnostic complexity and AI contribution, originally in a pricing and reimbursement context [15]. The present article does not use DACS as a payment instrument. Instead, it uses the four DACS dimensions—data complexity and diversity, disease complexity and severity, complexity of the clinical question, and degree of AI involvement—as an upstream characterization of AI-CDSS deployment profiles. The central hypothesis is that these deployment profiles can guide proportional outcome measurement: higher clinical stakes, greater AI involvement, more complex questions, and more heterogeneous data should trigger more stringent outcome-domain selection, telemetry requirements, governance safeguards, and validation burden.

The article is a *Hypothesis and Theory* contribution. It does not report a prospective validation study, pooled effects, or new patient-level data. Rather, it develops a conceptual and methodological framework that is intended to be testable in subsequent empirical work. The framework contributes four elements: (i) a six-domain outcome taxonomy for AI-CDSS; (ii) a comparison with existing AI evaluation and reporting frameworks; (iii) a DACS-informed outcome-domain mapping with an explicitly illustrative prioritization heuristic and threshold-sensitivity logic; and (iv) a four-step measurement framework with minimum telemetry requirements, clinical implementation guardrails, and LLM-specific extensions.

## 2 Methods

### 2.1 Overall approach

We developed a concept-driven structured narrative synthesis coupled to framework development. The objective was not to conduct a systematic review or estimate pooled effects, but to integrate concepts from clinical AI evaluation, clinical decision support, software measurement, health-services research, health economics, and AI governance into a testable conceptual model for AI-CDSS outcome measurement. The manuscript should therefore be interpreted as a theory-building and framework-development article rather than as empirical validation of DACS, the DACS-to-domain mapping, or the proposed threshold heuristic.

The work proceeded in five steps. First, we identified conceptual requirements for outcome-based AI-CDSS evaluation, including clinical relevance, exposure measurement, action linkage, attribution, auditability, governance, and measurement burden. Second, we reviewed existing AI evaluation and reporting frameworks to determine which aspects were already covered and which deployment-level measurement decisions remained under-specified. Third, we extended DACS from a reimbursement-oriented construct to a measurement-oriented characterization tool. Fourth, we derived a six-domain outcome taxonomy and a DACS-to-domain mapping. Fifth, we applied the framework to illustrative deployment archetypes to demonstrate how measurement priorities, telemetry requirements, and governance safeguards could differ by use case.

### 2.2 Structured narrative search and source selection

We used a concept-driven search strategy rather than an exhaustive systematic-review protocol. Searches were conducted across PubMed/MEDLINE and Google Scholar, complemented by targeted identification of methodological, regulatory, and clinical-informatics sources. Searches were organized around six search strands: (i) clinical AI/CDSS evaluation; (ii) outcome and value measurement; (iii) reporting and appraisal frameworks; (iv) governance and accountability; (v) clinical implementation risks; and (vi) LLM deployment. We included peer-reviewed empirical studies, reporting guidance, methodological frameworks, policy documents, and position papers that informed outcome measurement, attribution, implementation, monitoring, or governance of AI-CDSS.

Sources were eligible if they met at least one of the following criteria: (a) proposed or operationalized outcome metrics for clinical AI, CDSS, or digital health; (b) addressed clinical implementation risks such as alert fatigue, automation bias, workflow integration, or de-implementation; (c) specified measurement infrastructure, telemetry, auditability, or post-deployment monitoring requirements; (d) discussed reimbursement, procurement, value-based care, or economic evaluation relevant to AI-CDSS; or (e) provided regulatory or governance context relevant to deployed AI systems. We excluded purely technical benchmarking papers without implications for clinical workflow, outcome measurement, or governance, as well as model-development studies that did not address deployment or outcome evaluation.

The search was not designed to produce a PRISMA-conformant record flow, formal risk-of-bias assessment, or exhaustive evidence map. To improve auditability of the conceptual synthesis, Table 1 provides the search concepts, example executable strings, PubMed/MEDLINE record counts from a documented re-run of the example strings during revision (12 June 2026), and the role of each search strand in framework development. In a future systematic review or scoping review, these strings should be prospectively registered, database-specific syntax should be preserved, record counts should be logged at each stage, and screening reliability should be reported. In the present article, the search served to support conceptual saturation and framework construction.

**Table 1:** Search log for the concept-driven structured narrative synthesis.

Search strand	Example executable search string	PubMed yield	Role in framework development
Clinical AI/CDSS evaluation	(“clinical decision support” OR CDSS OR “clinical AI”) AND (evaluation OR “real-world evaluation” OR implementation OR monitoring)	9,459	Identified concepts for real-world evaluation, post-deployment monitoring, and implementation-sensitive outcome measurement.
Outcome and value measurement	(“clinical decision support” OR “digital health”) AND (outcomes OR “value-based care” OR “budget impact” OR “cost-effectiveness” OR “resource utilization”)	16,076	Informed clinical, operational, economic, and organizational outcome domains.
Reporting and appraisal frameworks	(CONSORT-AI OR SPIRIT-AI OR DECIDE-AI OR TRIPOD-AI OR PROBAST-AI OR TEHAI)	257	Used to position the proposed framework relative to existing reporting, trial, early evaluation, and prediction-model appraisal guidance.
Governance and accountability	(“artificial intelligence” AND healthcare AND (governance OR accountability OR “audit trail” OR procurement OR reimbursement OR “risk sharing”))	2,512	Informed telemetry, auditability, accountability, and downstream procurement/payment discussion.
Clinical implementation risks	(“clinical decision support” AND (“alert fatigue” OR override OR “automation bias” OR anchoring OR “de-implementation” OR workflow))	2,044	Informed clinical guardrails, alert-budget logic, override monitoring, automation-bias safeguards, and de-implementation criteria.
LLM deployment	(“large language model” OR LLM OR “foundation model”) AND (clinical OR healthcare) AND (documentation OR workflow OR evaluation OR governance OR cost)	4,214	Informed LLM-specific provenance, human mediation, documentation audit, latency, and compute-cost considerations.

The original searches used PubMed/MEDLINE and Google Scholar, complemented by targeted source identification; the yield column reports PubMed/MEDLINE record counts from a documented re-run of the example strings on 12 June 2026.

### 2.3 Thematic synthesis and framework construction

We extracted and organized concepts into six analytic categories: (1) outcome domains; (2) metric proximity and attribution; (3) exposure–action–outcome linkage; (4) telemetry and auditability; (5) governance and safety guardrails; and (6) LLM-specific measurement implications. Thematic synthesis was iterative and interpretive. Candidate concepts were retained when they were repeatedly relevant across clinical AI evaluation, CDSS implementation, software measurement, health-services research, or governance literatures, or when they addressed a known deployment failure mode such as alert fatigue, automation bias, model drift, or poor integration with existing quality infrastructure. During revision, references were rechecked for claim-source alignment; citations were removed or narrowed where the cited source did not directly support the operational claim.

The resulting framework was evaluated for internal coherence using four questions: (i) Does each DACS dimension plausibly change the minimum outcome domains that should be measured? (ii) Can the proposed metrics be linked to routinely collectible data sources? (iii) Does the telemetry schema support exposure–action–outcome linkage without mandating excessive data capture? (iv) Are governance safeguards included for clinically important failure modes, including alert burden, override behavior, automation bias, de-implementation, patient disclosure, and equity?

### 2.4 Use-case scoring and status of DACS ratings

DACS assigns ordinal scores from 1 to 5 across four dimensions: data complexity and diversity, disease complexity and severity, complexity of the clinical question, and degree of AI involvement [15]. For this article, DACS was used only as a deployment-characterization tool. The illustrative use-case profiles are not validation evidence. They were constructed to demonstrate how different deployment archetypes could trigger different measurement priorities. Two authors independently applied the published DACS criteria to each use case and then resolved differences through consensus. Because the number of illustrative ratings is small, we do not present inferential inter-rater statistics. Instead, Table 2 reports the consensus scores and explicitly labels them as illustrative. Future validation should quantify inter-rater reliability in a larger sample of AI-CDSS use cases using weighted kappa or intraclass correlation, depending on the final scoring protocol.

**Table 2:** Illustrative DACS profiles and resulting priority outcome domains.

Use case	Data	Severity	Question	AI use	Illustrative priority domains
Early sepsis detection	4	5	3	4	Clinical safety; operational alert burden; learning/governance; equity; economic impact. Mortality should be treated as distal and weakly attributable without strong adjustment.
Complex laboratory work-up support	3	3	4	3	Operational/resource; economic/organizational; experience; learning/governance; equity where diagnostic delays or access differ by subgroup.
LLM-assisted consultation and documentation	3	3	4	3	Operational documentation burden; experience and cognitive load; learning/governance; equity by language/literacy; economic compute and integration cost.
Imaging triage and worklist prioritization	4	4	3	4	Clinical time-to-treatment for critical findings; operational queue effects; governance/override; equity by scanner/site/protocol; economic capacity effects.

## 3 Results

### 3.1 Overview of framework outputs

**Figure 1:**
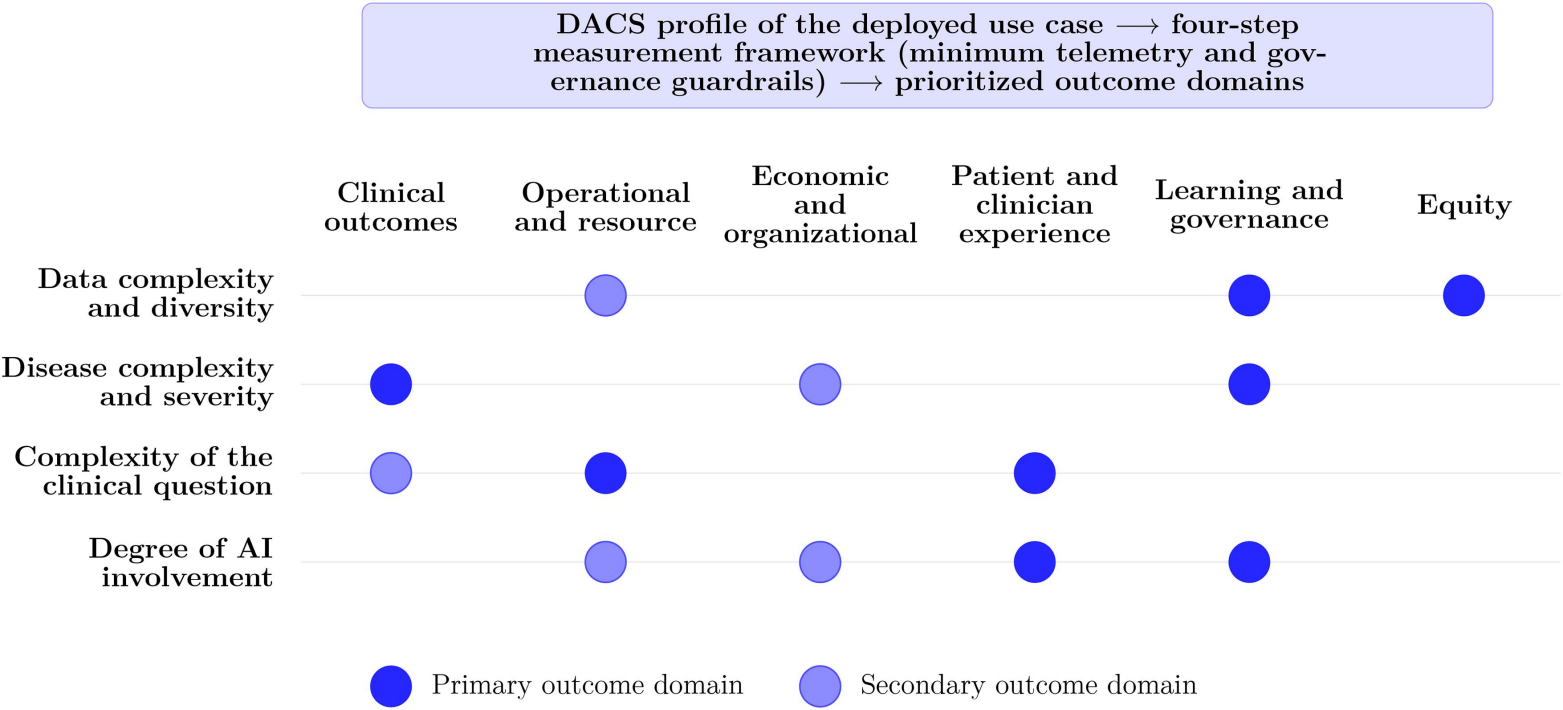
Preferential routing from DACS dimensions to outcome domains. Each deployed AI-CDSS use case is scored on the four DACS dimensions; the four-step measurement framework then prioritizes the marked outcome domains together with their minimum telemetry and governance guardrails. Dark circles denote outcome domains that are primarily sensitive to a given DACS dimension and light circles denote secondarily sensitive domains, consistent with the conceptual mapping in Table 3 and the illustrative use-case profiles in Table 2. The routing is hypothesis-generating and requires empirical validation.

The proposed framework has four outputs; Figure 1 summarizes how the four DACS dimensions, via the four-step measurement framework, preferentially route to the six outcome domains, consistent with the conceptual mapping in Table 3. First, the framework defines six outcome domains for AI-CDSS: clinical outcomes, operational and resource outcomes, economic and organizational outcomes, patient and clinician experience, learning and governance outcomes, and equity outcomes. Second, it clarifies the incremental role of this framework relative to existing evaluation and reporting guidance. Third, it maps DACS dimensions to outcome-domain priorities and proposes an explicitly illustrative threshold heuristic. Fourth, it specifies a four-step measurement process with minimum telemetry and clinical implementation guardrails.

**Table 3:** Conceptual mapping between DACS dimensions and outcome domains.

DACS dimension	Outcome domains most sensitive to this dimension
Data complexity and diversity	Higher scores imply heterogeneous, multimodal, longitudinal, or cross-system data. Measurement should emphasize linkage quality, source-data provenance, missingness, data drift, operational effects of data retrieval, and equity effects caused by uneven data completeness.
Disease complexity and severity	Higher scores increase the salience of clinical safety, escalation, risk adjustment, and distal outcomes. High severity should trigger clinical outcomes, safety endpoints, governance review, and fallback workflows.
Complexity of the clinical question	Higher scores indicate multi-step reasoning, differential diagnosis, or pathway selection. Measurement should emphasize intermediate pathway metrics, diagnostic cascade length, low-yield tests, structured differentials, clinician confidence, and attribution-ready process outcomes.
Degree of AI involvement	Higher scores increase potential benefit and potential harm. Measurement should emphasize oversight, override, escalation, auditability, automation bias, drift monitoring, user training, de-implementation criteria, and patient-facing disclosure where relevant.

### 3.2 Relationship to existing AI evaluation and reporting frameworks

Table 4 summarizes how the proposed framework relates to existing frameworks. The aim is not to replace reporting or evaluation guidance. Instead, the framework addresses a narrower operational question: given a specific AI-CDSS deployment profile, which outcome domains and telemetry elements should be prioritized so that evaluation, monitoring, and governance are proportionate to clinical stakes, AI involvement, question complexity, and data complexity?

**Table 4:** Relationship of the proposed framework to selected AI evaluation and reporting frameworks.

Framework	Primary purpose	Deployment workflow	Outcome-domain prioritization	Telemetry/attribution	Incremental role of the present framework
CONSORT-AI/SPIRIT-AI	Reporting of AI intervention trials and protocols	Requires description of intervention and human-AI interaction	Does not prescribe DACS-conditioned outcome priorities	Does not define minimum exposure–action–outcome telemetry	Complements trial reporting by specifying deployment-sensitive metric selection and telemetry classes.
DECIDE-AI	Early-stage clinical evaluation of decision-support systems	Strong attention to clinical setting and human factors	Broad evaluation orientation rather than DACS-specific prioritization	Does not provide a general telemetry schema for routine monitoring	Adds proportional outcome prioritization and audit-oriented telemetry for routine deployments.
TRIPOD+AI	Reporting prediction-model studies	Focuses on model development/validation reporting	Not designed for outcome-domain selection in deployed CDSS	Not designed as a workflow telemetry standard	Extends beyond prediction reporting toward real-world outcome and governance measurement.
PROBAST+AI	Risk-of-bias and applicability assessment for prediction models	Addresses applicability but not workflow governance	Not an outcome prioritization tool	Not an implementation telemetry schema	Complements model appraisal with post-deployment outcome and action linkage.
TEHAI	Translational evaluation of healthcare AI	Broad translational and implementation orientation	Multi-domain evaluation, but not linked to DACS profiles	Does not operationalize a minimum telemetry model	Adds a deployable heuristic linking use-case complexity to measurement requirements.
Proposed framework	DACS-informed outcome measurement and governance	Central: measures deployed workflow exposure, action, and outcomes	Central: domains prioritized by DACS profile and clinical context	Central: minimum telemetry schema and audit logic	Provides a testable bridge from AI-CDSS use-case characterization to proportional outcome measurement.

### 3.3 Outcome domains for AI-CDSS

Table 5 presents the six outcome domains and exemplar metrics. We separate learning/governance from equity because these are related but distinct concerns. Learning and governance focus on monitoring, drift, feedback loops, update control, overrides, auditability, and de-implementation. Equity focuses on differential access, performance, burden, and downstream effects across patient groups, sites, languages, literacy levels, and care contexts.

**Table 5:** Outcome domains and exemplar metrics for AI-CDSS, with attribution considerations.

Outcome domain	Exemplar metrics and attribution considerations
Clinical outcomes	Time to diagnosis; time to treatment; diagnostic yield; guideline-concordant care; diagnostic error where measurable; adverse events; avoidable complications; readmissions; disease control. Distal endpoints such as mortality should be labelled as weakly attributable unless supported by strong designs and risk adjustment.
Operational and resource outcomes	Alert burden; alert-to-action ratio; acknowledgment and override rates; time spent on documentation or information retrieval; diagnostic cascade length; duplicate or low-yield tests; turnaround time; throughput; queue effects; length of stay; downtime affecting availability.
Economic and organizational outcomes	Direct cost per episode; total cost of ownership; integration and maintenance costs; cost-center shifts; staffing and skill-mix effects; overtime or on-call burden; DRG or contribution-margin effects where applicable; documentation completeness; audit risk; certification-relevant metrics; budget impact.
Patient and clinician experience	PROMs and PREMs; patient understanding; reassurance; trust; perceived fairness and transparency; clinician cognitive load; usability; perceived autonomy; training burden; shared decision-making; disclosure-related trust and adherence.
Learning and governance outcomes	Data completeness; input-data drift; calibration drift; update history; model/version traceability; override patterns; escalation pathways; governance actions; de-implementation triggers; fallback workflow readiness; incident reports and safety reviews.
Equity outcomes	Subgroup-stratified false-negative and false-positive rates; differential alert burden; differential access to AI-supported workflows; subgroup-specific time to action; language and literacy effects; differential override rates; fairness-aware risk adjustment; monitoring for allocative and representational harms.

For each metric, implementers should specify proximity and attributability. Proximal metrics, such as exposure, alert acknowledgment, override, time to action, documentation time, or test-order changes, are usually more attributable to AI-CDSS. Intermediate metrics, such as diagnostic cascade length or time to treatment, require stronger linkage and contextual adjustment. Distal metrics, such as mortality, readmission, total cost, or long-term disease control, may be clinically important but should be interpreted as weakly attributable unless supported by rigorous designs, risk adjustment, and sensitivity analyses.

### 3.4 DACS as a measurement-oriented characterization tool

DACS characterizes diagnostic AI use cases along four dimensions: data complexity and diversity, disease complexity and severity, complexity of the clinical question, and degree of AI involvement [15]. In prior work, DACS was linked to reimbursement and pricing considerations. In the present framework, DACS is repositioned upstream of reimbursement as a measurement-design tool. Its purpose is to help determine which outcomes should be measured, how strongly attribution must be supported, what telemetry is required, and which governance safeguards should be mandatory.

Severity alone would not be sufficient because low-severity but highly workflow-integrated systems may have substantial operational, equity, or workload effects. AI involvement alone would not be sufficient because a highly autonomous low-stakes documentation assistant and a high-stakes sepsis alert raise different safety and governance requirements. Regulatory classification is also insufficiently granular for outcome-domain prioritization. DACS is therefore used as a multi-dimensional heuristic that combines clinical stakes, data complexity, reasoning complexity, and AI involvement. This rationale does not imply that DACS is validated or uniquely optimal. The DACS-to-domain mapping should be treated as a testable hypothesis and compared empirically with simpler alternatives such as severity-only, AI-involvement-only, or independent risk-classification approaches.

### 3.5 DACS-to-domain mapping and prioritization heuristic

Table 3 provides the conceptual DACS-to-domain mapping. The mapping indicates domains that are plausibly sensitive to each DACS dimension. It is not a fixed metric set and should not be interpreted as validated. It is intended to structure discussion among clinicians, informaticians, quality-management teams, procurement stakeholders, and governance bodies.

The operational heuristic uses scores of 4–5 as illustrative high-score triggers because they correspond to the upper two ordinal categories. These thresholds are not empirically calibrated. They are included to make the framework implementable and testable. Implementers should adapt thresholds to local clinical risk, governance maturity, data quality, and measurement burden.

- **Severity** ≥4: clinical outcomes, safety endpoints, risk adjustment, and explicit escalation/fallback pathways should be mandatory.
- **AI involvement** ≥4: governance and learning metrics should be mandatory, including override tracking, escalation traces, drift monitoring, version control, and de-implementation criteria.
- **Data complexity** ≥4: exposure logging, source-data provenance, linkage quality, missingness monitoring, and input-data drift should be prerequisites before interpreting distal outcomes.
- **Question complexity** ≥4: intermediate pathway metrics should be included to strengthen attribution, for example diagnostic cascade length, low-yield test rates, structured differential completeness, or time to definitive work-up.
- **LLM deployments with high involvement or high question complexity:** prompt/output provenance policy, model/version identifiers, human mediation measures, documentation-quality audits, and latency/compute metering should be added.

Table 6 illustrates how threshold choice changes measurement burden. A lower threshold increases sensitivity to potential risks but may generate excessive measurement burden; a higher threshold reduces burden but may under-measure clinically relevant effects. This threshold-sensitivity logic should be empirically calibrated in future deployments.

**Table 6:** Threshold-sensitivity logic for illustrative DACS triggers.

Trigger rule	Expected effect	Interpretation
Score ≥3	High sensitivity; many domains triggered; greater telemetry and governance burden	Useful in early pilots or high-uncertainty deployments where under-measurement is more concerning than measurement burden.
Score ≥4	Balanced illustrative default; upper two ordinal categories trigger minimum requirements	Proposed as a pragmatic starting convention, not as a validated threshold. Requires local adaptation and prospective calibration.
Score =5	High specificity; fewer domains triggered; lower measurement burden	Useful where resources are limited but risks under-measuring important effects near the threshold.
No numeric trigger	Fully deliberative domain selection	Avoids false precision but may reduce consistency, reproducibility, and auditability across deployments.

### 3.6 A four-step measurement framework

The framework operationalizes outcome measurement through four steps.

**Step 1: Characterize the deployed use case.** Stakeholders score the deployed workflow rather than the abstract model. The same foundation model may require different measurement priorities when used for triage, documentation, patient messaging, diagnostic work-up, or coding support.

**Step 2: Select priority outcome domains.** Based on DACS profile, clinical context, and local governance requirements, stakeholders select a limited set of priority domains. A typical deployment should not attempt to measure all possible outcomes. Instead, it should combine proximal metrics for attribution, intermediate metrics for pathway relevance, and selected distal metrics where clinically necessary.

**Step 3: Define metrics with auditable exposure–action–outcome linkage.** Metric specifications should include denominator, eligibility criteria, exposure definition, action definition, outcome window, data sources, linkage keys, confounders, risk adjustment, and audit rules. Metrics should distinguish technical availability from actual workflow exposure and should capture clinician response where possible.

**Step 4: Embed metrics into governance and quality infrastructure.** Metrics should be integrated into monitoring dashboards, quality-improvement cycles, clinical governance committees, patient-safety processes, and de-implementation procedures. This integration should follow established CDSS implementation principles: decision support must be embedded at the point of decision, fit existing workflow, and be monitored after deployment rather than treated as a static technical artifact [16–18]. Outcome-linked procurement, reimbursement, or contracting should be considered only where endpoints are sufficiently attributable, auditable, and protected against gaming [8, 19].

### 3.7 Minimum telemetry for auditable measurement

Table 7 specifies the minimum telemetry classes required for auditable AI-CDSS outcome measurement. The schema is technology-agnostic and can be implemented through electronic health record audit logs, order-entry systems, CDS hooks, middleware events, RIS/PACS events, laboratory information systems, vendor logs, or governance logs. The schema is intentionally framed as a minimum information model rather than a mandated data model. Implementations should follow data-minimization principles, role-based access, separation of identifiers, retention limits, and local privacy requirements.

**Table 7:** Minimum telemetry for auditable outcome measurement in AI-CDSS.

Telemetry element	Operational definition and examples
Eligibility	Defines the denominator: patients or encounters eligible for AI-CDSS under the protocol. Example fields: inclusion/exclusion flags, unit, encounter type, baseline risk strata.
Availability	Whether AI-CDSS was technically available at the decision point. Example fields: uptime, active version, endpoint status, integration status, downtime events.
Workflow exposure	Whether AI output was presented, when, where, to whom, and in what form. Example fields: exposure event ID, alert shown, score displayed, explanation shown, UI placement, timestamp, user role.
Action	Measurable response in the workflow. Example fields: acknowledgment, override, order placed, escalation, pathway selected, documentation accepted/edited, time to action.
Outcome window	Follow-up horizon and linkage rules. Example fields: 6 h, 24 h, 30 day windows; censoring rules; competing events; attribution hierarchy.
Confounders and risk adjustment	Variables needed to interpret outcome changes. Example fields: baseline severity, comorbidities, staffing, case mix, concurrent interventions, seasonality, site effects.
Governance and audit trail	Linkage keys and review traces. Example fields: encounter linkage key, exposure ID, user/session ID, version ID, immutable timestamp, override reason, escalation review, de-implementation trigger.
LLM provenance and metering	Model/version ID, retrieval-source ID, prompt/output provenance policy, redaction status, token/compute use, latency, review result, documentation-quality audit result where applicable.

Telemetry supports plausible attribution by making exposure and response observable. It does not by itself establish causality. For causal inference, telemetry should be combined with appropriate designs such as randomized trials, stepped-wedge implementation, controlled before–after designs, interrupted time series, or prospective registry-based evaluation [20, 21].

### 3.8 Clinical implementation guardrails

The framework requires clinical guardrails because outcome measurement can fail if implementation risks are reduced to generic monitoring. Five guardrails are particularly important.

**Alert burden and alert-to-action ratio.** For alerting systems, pre-deployment evaluation should define an alert budget, such as expected alerts per clinician per shift, alerts per patient-day, and acceptable alert-to-action ratios. Post-deployment monitoring should include alert burden, acknowledgment, override, escalation, and time-to-action. A sustained increase in alert burden without proportional clinical action should trigger re-tuning, workflow redesign, or de-implementation review [6, 22–24].

**Override patterns by user group.** Override rates should be stratified by specialty, ward, seniority, and professional role where governance permits. A senior specialist overriding most alerts and a junior physician accepting most alerts may indicate different issues: expertise-dependent calibration, training needs, alert specificity problems, or inappropriate reliance [22, 24].

**Automation bias and diagnostic anchoring.** When AI-CDSS presents a top-ranked suggestion, differential diagnosis, or risk score, evaluation should consider anchoring and automation bias. Possible measurement strategies include structured differential elicitation before AI exposure, blinded second-reader designs, randomization of explanation timing, and monitoring of premature diagnostic closure [25, 26].

**Skill atrophy and educational effects.** In academic centers, AI-CDSS may influence training of junior clinicians. Measurement should include educational-impact indicators where relevant, such as independent reasoning documentation, supervision patterns, and periodic non-AI case review. This is especially important when AI changes the amount of independent diagnostic reasoning performed by trainees before supervisory review.

**De-implementation and fallback workflows.** Governance plans should define degradation thresholds, authority to suspend the system, safe fallback workflows, user communication, retraining on non-AI pathways, and revalidation after major model, interface, or data-pipeline changes. De-implementation should be treated as a normal safety function rather than as a failure of innovation [17, 18].

### 3.9 LLM-specific measurement considerations

LLM-based AI-CDSS often produces workflow-mediated effects rather than direct clinical actions. Outcome measurement should therefore emphasize documentation time, note quality, communication quality, clinician cognitive load, patient understanding, latency, compute cost, and provenance. Prompt and output logging may be clinically and legally sensitive; therefore, the framework recommends a provenance policy rather than universal full-content logging. Depending on local governance, this may include hashed prompt identifiers, redacted prompt/output samples, model and version identifiers, retrieval-source identifiers, user role, timestamp, and structured review results.

For LLM-assisted documentation, normalized edit similarity can be used as one workload and human-mediation indicator, but it should never be interpreted as a clinical correctness, safety, or documentation-quality metric. A note accepted verbatim may contain an error, while a heavily edited note may be clinically superior. If used, edit similarity should be paired with semantic equivalence checks, entity/negation/dosage-change detection, sampled clinical audits for omissions and hallucinations, and structured reviewer ratings. To avoid Goodhart-type effects, edit similarity should not be used as a standalone target for performance management or reimbursement [19].

### 3.10 Integration with existing hospital quality and regulatory infrastructure

The framework should not create a parallel measurement universe detached from hospital quality systems. AI-CDSS metrics should be mapped to existing quality-management and patient-safety structures, including morbidity and mortality conferences, incident reporting systems, clinical risk management, certification metrics, center-specific quality indicators, and national reporting requirements where applicable. In German and European settings, this may include alignment with IQTIG-related quality indicators, G-BA requirements, DKG center-certification metrics, clinical risk-management processes, the Medical Device Regulation (MDR), and the EU AI Act where relevant [27, 28].

Patient-facing disclosure should also be considered. Where AI materially influences diagnostic reasoning, communication, triage, or documentation, governance should specify whether and how AI involvement is documented, disclosed, and explained. Disclosure practices may affect trust, adherence, satisfaction, and perceived fairness, and should therefore be part of the patient and clinician experience domain rather than treated as a purely legal issue.

### 3.11 Illustrative use cases

#### 3.11.1 Use case 1: early sepsis detection in hospital wards

Sepsis alerting typically has high disease severity and high AI involvement. Priority metrics should include time to recognition, time to antibiotics, escalation timeliness, alert burden, alert-to-action ratio, override patterns, ICU transfer, length of stay, and mortality only as a distal endpoint requiring strong adjustment. The sepsis use case also requires monitoring for case-mix shifts, ward-specific calibration, immunosuppressed or hematologic-malignancy populations, seasonal effects, and training effects related to sepsis bundles [6].

#### 3.11.2 Use case 2: diagnostic work-up support in laboratory medicine

Laboratory work-up support often affects diagnostic cascades rather than immediate mortality. Priority metrics should include redundant tests, low-yield test rates, guideline-concordant cascades, time to definitive diagnosis, test turnaround, downstream referrals, cost per diagnostic episode, and clinician confidence. Attribution is strengthened by measuring intermediate pathway metrics rather than relying on distal outcomes.

#### 3.11.3 Use case 3: LLM-assisted consultation and documentation

LLM-assisted documentation and consultation support should emphasize documentation time, time to sign, information retrieval, note completeness, clinician cognitive load, patient understanding, prompt/output provenance policy, version tracking, human mediation, sampled documentation-quality audits, latency, and compute cost. Clinical outcomes may be relevant but are often indirectly attributable.

#### 3.11.4 Use case 4: imaging triage and worklist prioritization

AI-supported radiology triage illustrates a deployment in which attribution differs from laboratory or ward-based CDSS. Priority metrics may include time from image acquisition to alert, time to radiologist review, time to report for critical findings, downstream time to treatment, false-negative review, interruption burden, reprioritization effects on non-flagged cases, subgroup performance by scanner/site/protocol, and PACS/RIS telemetry linkage. This use case highlights that high workflow integration may generate value or harm through prioritization, queue dynamics, and interruption effects even when the final diagnostic decision remains with the radiologist.

## 4 Discussion

### 4.1 Principal contribution

This article proposes a testable conceptual framework for linking diagnostic AI contribution to outcome measurement in AI-CDSS. The contribution is not empirical validation. Rather, the framework offers a structured model for deciding which outcome domains should be prioritized, what telemetry is minimally required, and which governance safeguards should accompany different deployment profiles. Its distinctive elements are DACS-conditioned prioritization, exposure–action–outcome telemetry, explicit attention to clinical implementation guardrails, and LLM-specific measurement extensions.

### 4.2 Interpretation of the DACS-to-domain mapping

The DACS-to-domain mapping should be interpreted as a hypothesis-generating model. It is internally plausible because the four DACS dimensions correspond to clinically meaningful sources of measurement burden and governance risk: data complexity affects linkage and provenance; disease severity affects safety and risk adjustment; question complexity affects pathway attribution; and AI involvement affects oversight, override, and de-implementation requirements. Nevertheless, the mapping remains expert-derived and requires external validation. Future studies should compare the DACS-informed heuristic with simpler alternatives, including severity-only, AI-involvement-only, and regulatory-risk approaches.

### 4.3 Validation roadmap

Empirical validation should proceed in four stages. First, inter-rater reliability of DACS scoring should be tested across a diverse sample of AI-CDSS use cases with raters from clinical, informatics, quality-management, and procurement backgrounds. Second, the DACS-to-domain mapping should be evaluated through Delphi or structured expert elicitation to determine whether experts agree that specific DACS profiles imply specific measurement priorities. Third, prospective deployment studies should calibrate the threshold heuristic by comparing outcomes and measurement burden under different trigger rules, such as thresholds of 3, 4, or 5. Fourth, multi-site studies should evaluate whether standardized telemetry improves attribution, monitoring, safety review, and procurement decisions.

### 4.4 Implications for clinical governance and procurement

For clinical governance, the framework supports proportional monitoring: high-stakes and high-involvement systems require stronger safety metrics, override analysis, de-implementation criteria, and fallback workflows. For hospital leadership, the framework translates AI-CDSS value into metrics that can be connected to operational and financial decision-making, such as length-of-stay effects relative to reimbursement structures, cost-center shifts, staffing needs, documentation completeness, audit risk, certification-relevant indicators, and total cost of ownership. For vendors and payers, the framework clarifies that outcome-linked payment or risk-sharing is plausible only when endpoints are attributable, auditable, balanced against gaming, and protected against equity harms.

### 4.5 Reflexivity and conflict-of-interest considerations

Two authors are employed by a company that develops diagnostic decision-support software, and DACS originated in prior work by the authors. This creates a potential framing interest: a framework that emphasizes diagnostic complexity and AI contribution could be perceived as supporting procurement or reimbursement arguments favorable to vendors. We address this in three ways. First, the present manuscript explicitly does not propose payment levels, tariffs, or reimbursement claims. Second, DACS is positioned as a candidate characterization tool rather than as a validated standard. Third, the framework calls for external validation, comparison with simpler alternatives, and transparent governance safeguards before DACS-informed measurement is used in procurement or contracting. These safeguards are necessary to avoid circularity and to ensure that outcome measurement remains clinically and publicly accountable.

### 4.6 Limitations

This work is conceptual and should not be interpreted as empirical validation. The structured narrative synthesis was not a systematic review, did not include PRISMA record accounting, and did not conduct formal risk-of-bias assessment. The DACS-to-domain mapping is expert-derived and hypothesis-generating. The illustrative threshold of 4 is not empirically calibrated. The use cases demonstrate application logic but do not prove generalizability. Some proposed metrics, particularly distal clinical and economic endpoints, may be weakly attributable without rigorous designs and risk adjustment. Finally, telemetry requirements may vary by legal context, technical infrastructure, and organizational governance maturity.

## 5 Conclusions

AI-CDSS evaluation must move beyond model-centric performance toward auditable measurement of real-world clinical, operational, economic, experiential, learning, governance, and equity outcomes. This article proposes a testable conceptual framework that links DACS-based use-case characterization to outcome-domain prioritization and minimum telemetry. The framework is intended to support proportional measurement design, clinical governance, and future empirical validation, not to serve as a validated reimbursement instrument.

Future work should validate inter-rater reliability of DACS scoring, test the DACS-to-domain mapping through expert elicitation and prospective deployments, calibrate threshold heuristics, and evaluate whether standardized exposure–action–outcome telemetry improves attribution and governance. As LLM-based and generalist AI systems expand the scope of clinical decision support, structured and auditable measurement frameworks will be necessary to support accountable adoption while protecting safety, clinical expertise, patient trust, and equity.

## Data Availability

The original contributions presented in the study are included in the article/Supplementary Material, further inquiries can be directed to the corresponding author.
